# SARS-CoV-2 Variants and Their Impact on Pediatric COVID-19: Clinical Manifestations and Hematological Profiles

**DOI:** 10.3390/diseases13020048

**Published:** 2025-02-06

**Authors:** Konstantinos Paris Trempelis, Chrysoula Kosmeri, Panagiotis Kalavas, Fani Ladomenou, Ekaterini Siomou, Alexandros Makis

**Affiliations:** 1 Faculty of Medicine, School of Health Sciences, University of Ioannina, 45500 Ioannina, Greece; ktrempelis@gmail.com (K.P.T.); faniladomenou@uoi.gr (F.L.); eksiomou@uoi.gr (E.S.); 2 Department of Pediatrics, University Hospital of Ioannina, 45500 Ioannina, Greece

**Keywords:** SARS-CoV-2, COVID-19, strain, children, pediatric, outcomes, clinical manifestations, hematological findings

## Abstract

Background: The aim of this study was to analyze data on pediatric cases of COVID-19 admitted to a tertiary referral hospital in northwest Greece. Methods: A retrospective analysis was conducted on the most common clinical manifestations and laboratory findings, stratified by age group and SARS-CoV-2 strain. Results: A total of 254 children were hospitalized, with a mean age of 4.5 years. Underlying conditions were present in 10.2% of cases; two children required pediatric intensive care unit (PICU) admission, and one child died. The most common hematological manifestations, in general, were neutropenia (30%) and lymphopenia (23%), whereas the findings varied when the children were stratified by age group. Eight children developed multisystem inflammatory syndrome (MIS-C), with the most common findings being anemia (75%), lymphopenia (50%), and thrombocytopenia (25%). Analysis of the SARS-CoV-2 strains revealed the proportions of the dominant strain over time. Fever was the predominant symptom across all strains, particularly in the Omicron group, which also had a high incidence of gastrointestinal symptoms. The longest hospital admission occurred in children with the Omicron strain, followed by the Wuhan, Alpha, and Delta strains. Conclusions: Fever was the most consistent symptom across all age groups and virus strains. The most common hematological manifestations were neutropenia (30%) and lymphopenia (23%). The Omicron strain was associated with the longest hospital stay.

## 1. Introduction

Severe acute respiratory syndrome coronavirus 2 (SARS-CoV-2) first appeared in late 2019 in Wuhan, a city located in Hubei Province, China [1]. It rapidly spread across the globe, and, on 11 March 2020, the World Health Organization (WHO) declared COVID-19 a pandemic [2]. Almost three years later, on 5 May 2023, the WHO announced that it was no longer a public health emergency of international concern (PHEIC) [3]. Despite the relative decline, new mutations continue to emerge, forcing healthcare systems worldwide to remain vigilant [4]. To date, extensive research has been conducted to better understand the pathogenesis and effects of the virus in both adult and pediatric populations.

SARS-CoV-2 uses the angiotensin-converting enzyme 2 (ACE2) receptor to enter host cells [5]. The process is facilitated by the virus’s spike protein, which binds to ACE2. Variants such as the original strain and Delta require the TMPRSS2 enzyme to cleave the spike protein, enabling membrane fusion and efficient viral entry into cells [6]. In contrast, Omicron-lineage variants primarily utilize an alternative entry mechanism involving cathepsin-dependent endocytosis and rely much less on TMPRSS2. This shift in entry pathways enables Omicron variants to replicate more efficiently in the upper respiratory tract, where TMPRSS2 expression is significantly lower than that in the lungs [7]. These differences in entry mechanisms contribute to the distinct replication patterns and clinical presentations associated with different SARS-CoV-2 variants.

According to the WHO, more than 776 million infections have been recorded worldwide, with at least 7 million deaths reported [8]. In Greece, the total infections have exceeded 5,500,000, and deaths have surpassed 35,000 [8,9].

For the pediatric population, the data are more encouraging. Since the beginning of the pandemic, it has been widely recognized that SARS-CoV-2 infection in children is mostly asymptomatic or mildly symptomatic [10,11]. At the start of the pandemic, most COVID-19 cases in children were linked to household exposure, with adults typically being the primary source of infection [12]. Although the amount of SARS-CoV-2 virus shed by individuals can vary, children can have viral loads in their nasopharynx that are comparable with or even exceed those of adults [13]. Children, regardless of age, are capable of transmitting the virus, even if they do not show symptoms [13]. While children with underlying health conditions are at greater risk for severe illness, including hospitalization, intensive care, or death, there is limited conclusive evidence linking specific conditions to more severe disease outcomes [14]. Nevertheless, the mortality rate in children remains below 1% [15].

However, given the high prevalence of the virus and the potential comorbidities in children, cases of severe disease and multisystem inflammatory syndrome in children (MIS-C) have been observed. MIS-C is a rare complication of COVID-19, marked by significant cardiovascular involvement, gastrointestinal symptoms, and mucocutaneous manifestations [16]. In 2023, the Centers for Disease Control and Prevention (CDC) estimated the incidence of MIS-C to be 0.11 cases per million person-months [17]. There has been noted a decline in cases of MIS-C due to widespread exposure to SARS-CoV-2, particularly to the Omicron variant, and increased vaccination rates [18,19]. Evidence indicates that MIS-C is both less common and less severe with the Omicron variant than with earlier strains [20].

Despite these findings, the data regarding the pediatric population are significantly less comprehensive compared with those for adults. Therefore, the aim of this study was to identify and record the clinical manifestations of hospitalized children with COVID-19 in a tertiary referral hospital in northwest Greece and to analyze their hematological data by age group and the prevailing variant of the virus during each period.

## 2. Materials and Methods

### 2.1. Patient Population

This retrospective study collected and analyzed data from children hospitalized in the Infectious Diseases Unit of a tertiary hospital in northwestern Greece during the COVID-19 pandemic. The Children’s Infectious Diseases Unit of the University General Hospital of Ioannina served as a referral center for COVID-19 cases, treating children who were admitted either through the Emergency Department or transferred from secondary hospitals in Northwestern Greece. In total, 254 children aged 0–16 years were admitted to the unit from the beginning of the pandemic, with the first case recorded in July 2020 and the last in March 2023.

### 2.2. Data Retrieval

The process of data extraction for this study was carried out using the hospital’s comprehensive information system. The patients included in this study were those who met specific admission criteria. These criteria required hospitalization in the Department of Pediatric Infectious Diseases at the hospital, with COVID-19 being the primary discharge diagnosis. The diagnosis of COVID-19 was coded according to the International Classification of Diseases, 10th Revision (ICD-10), under the code U07-1, which indicates confirmed cases of SARS-CoV-2 infection. Confirmation of the SARS-CoV-2 virus in all cases was performed using a commercial multiplex RT-qPCR (reverse-transcription quantitative real-time PCR) assay on nasopharyngeal swab specimens, a highly sensitive and specific diagnostic tool.

### 2.3. Demographic Data

For each patient, demographic information, including age and sex, was recorded. Additionally, a detailed personal medical history was documented, focusing on the presence of any pre-existing or underlying medical conditions. The length of each patient’s hospitalization was also noted. The clinical manifestations of the disease were thoroughly documented, encompassing a wide range of symptoms. These included respiratory symptoms, gastrointestinal complaints, chest pain, and neurological symptoms indicative of central nervous system involvement. Complications arising from the disease, such as myocarditis and pneumonia, were also carefully noted.

### 2.4. Laboratory and Imaging Data

Laboratory data were collected both at the time of admission and throughout the hospitalization period. These laboratory parameters included a complete blood count, measurements of serum creatinine and urea levels, liver function tests, and coagulation profiles, specifically prothrombin time (PT) and activated partial thromboplastin time (aPTT). Additionally, the levels of d-dimers, c-reactive protein (CRP), high-sensitivity troponin, and ferritin were measured to assess the extent of systemic inflammation and potential organ involvement. Further diagnostic evaluations included findings from chest X-rays and electrocardiograms, which provided insight into respiratory and cardiac involvement.

Details regarding the treatments administered to patients during their hospitalization were also systematically recorded. This included the use of oxygen therapy, inhalation medications, antibiotics, and any other therapeutic interventions. The overall clinical outcome for each patient was documented, providing valuable data on recovery or disease progression. Cases that met the criteria for multisystem inflammatory syndrome in children (MIS-C) were also included in this study, allowing for a broader understanding of the disease spectrum.

### 2.5. Data Analysis

Following data collection, the clinical and laboratory manifestations, as well as the outcomes of the disease, were analyzed and compared across different age groups. To evaluate the impact of viral evolution, the study period was divided into distinct sub-periods aligned with the predominant SARS-CoV-2 variants circulating during each timeframe, as identified through data and reports from the Hellenic National Public Health Organization (EODY). The variant associated with each child was inferred based on the timing of viral acquisition. Comparisons were then made to evaluate differences in clinical presentations, hematological parameters, and outcomes associated with each dominant virus strain during its respective period of prevalence.

### 2.6. Statistical Analysis

Continuous data are reported as mean ± SD or median (range), while categorical variables are expressed as numbers (%). The Kolmogorov–Smirnov Z test was used to assess the normality of continuous variables that were found to deviate from a normal distribution. Comparisons across the groups of viral strains were conducted using the Kruskal–Wallis test. Relationships between the categorical variables were analyzed with the chi-square test. Spearman correlation coefficients were calculated to explore associations between hematological parameters and hospitalization duration. Multiple regression analysis was performed to identify independent predictors of hospitalization duration, ensuring that assumptions of normality, linearity, multicollinearity, and homoscedasticity were met. Covariates with *p*-values < 0.10 in the Spearman correlation analyses were included in the multivariate regression models. All *p*-values were two-sided, with *p* < 0.05 considered statistically significant. Statistical analyses were conducted using SPSS software (version 29.0; SPSS Inc., Chicago, IL, USA).

## 3. Results

In total, 254 children were hospitalized during the study period, ranging in age from 5 days to 16 years, with a mean age of 4.5 years. Of these, 55% (n = 139) were boys. The median duration of hospitalization was 4.4 days (SD ± 3.4 days). A total of 10% of the children (n = 26) had underlying diseases, including type I diabetes mellitus, epilepsy, juvenile idiopathic arthritis, congenital heart disease, congenital enteropathy, history of prematurity, holoprosencephaly with gastrostomy, Sanfilippo metabolic syndrome, β-thalassemia, sickle cell anemia, hereditary spherocytosis, and cystic fibrosis.

In the study population, the most frequently observed clinical manifestation was fever, which affected a substantial majority of children (79%), followed by decreased feeding or poor oral intake, reported in 41% of cases. Gastrointestinal symptoms, including diarrhea, vomiting, and abdominal discomfort, were noted in 39% of the children, while respiratory symptoms such as cough were present in 36%. Additionally, symptoms related to the central nervous system, such as headache and febrile seizures, were documented in 18% of the cases.

Regarding the hematological findings, the majority of children demonstrated normal levels of white blood cells, hemoglobin, and platelets upon laboratory evaluation. However, specific hematological abnormalities were observed in a subset of patients. Neutropenia was seen in 30% of the children, while lymphopenia was observed in 23%. Coagulation abnormalities were largely absent in this population, with no significant disorders detected. Only 2% of the children exhibited a mild and transient prolongation of activated partial thromboplastin time (aPTT), and elevated D-dimer levels (greater than 1) were found in 15% of cases. Elevated CRP levels were observed in 45 children, accounting for 18% of the study population. Transaminasemia, indicating liver enzyme elevation, was present in 72 children, or 28% of cases. Clinical examination and imaging studies, such as chest X-rays, revealed pneumonia in six children (2%), while, in most cases, the chest X-ray was normal or had non-specific findings indicative of viral infection. Myocarditis, a rare but serious cardiac complication, was diagnosed in one child. In terms of treatment, 10 children (4%) required oxygen therapy to address hypoxemia, while 16 children (6%) were treated with inhaled salbutamol to alleviate bronchoconstriction. Additionally, corticosteroid therapy was administered to 18 children, representing 7% of the study group.

There were eight cases of MIS-C (3%) in children aged 2.5 months to 14 years, with a median hospital stay of 14 days. Among this group, lymphopenia was noted in 50% of cases, anemia in 75%, and thrombocytopenia in 25%. The treatment for MIS-C included intravenous immunoglobulin and aspirin therapy (used in three children). All the children had a full recovery without major complications.

Two children in this study required admission to the intensive care unit (ICU). The first was a previously healthy child who developed lung atelectasis and pleural effusion in the context of multisystem hyperinflammatory syndrome. The second child, who had an underlying metabolic condition known as Sanfilippo syndrome, unfortunately succumbed to the disease.

In the multivariate analysis, white blood cell count, CRP levels, and age were not identified as independent risk factors for hospitalization duration (R^2^ = 0.031, *p* = 0.39). Due to the limited number of children requiring ICU care or experiencing severe outcomes such as death, it was not feasible to perform correlations or multiple regression analyses to identify potential risk factors for these adverse outcomes.

### 3.1. Analysis by Age Groups

The demographic characteristics of the children, alongside the presence of underlying diseases, are shown in Table 1. The presence of underlying diseases was more common in children aged 1 to 11 years old. The mean duration of hospitalization did not differ among age groups.

The most common clinical manifestations in different age groups are shown in Table 2. Fever was the most common symptom across all age groups. Gastrointestinal symptoms were also common.

In infants and children up to 5 years of age, the most common laboratory manifestation was neutropenia (28%), while, in children older than 6 years and in adolescents, lymphopenia was more frequent (42–51%). Leukopenia was significantly less frequent in the age group of 1–5 years than in the other age groups. Thrombocytosis was observed in 17% of infants. The laboratory findings by age group are shown in detail in Table 3.

### 3.2. Analysis by SARS-CoV-2 Strains

**Figure 1:**
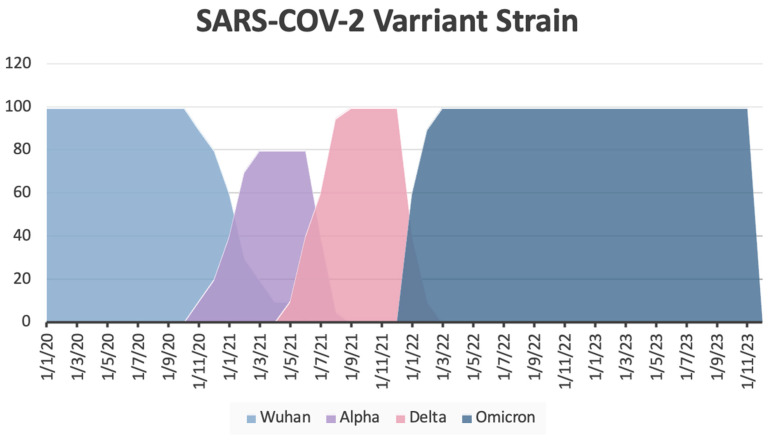
SARS-CoV-2 variant strain by time period in Greece (based on data from the Hellenic National Public Health Organization) [21].

An analysis of the SARS-CoV-2 subtypes/mutations prevailing in Greece during the pandemic showed that 2% of cases involved the Wuhan variant, 9% the Alpha variant, 17% the Delta variant, and 71% the Omicron variant (Figure 1).

Fever was the most common clinical finding in all variants, particularly Omicron (91%), for which there was also an increased incidence of gastrointestinal symptoms (43%). The median length of hospital stay during Omicron’s prevalence was 10 days, followed by Wuhan (9 days), Alpha (5 days), and Delta (4 days). There was a statistically significant difference in the duration of hospitalization between the two strains of Omicron and Wuhan compared with that for the Alpha and Delta strains (*p* = 0.024) (Table 4).

The most common clinical manifestations and laboratory findings based on the period of prevalence of each virus strain are shown in Table 4. The Kruskal–Wallis test revealed no statistically significant differences among the white blood cell count, neutrophil count, lymphocyte count, CRP levels, and age among different groups of viral strains.

## 4. Discussion

In this retrospective study of hospitalized children with COVID-19 in a tertiary referral hospital in Northwestern Greece, fever was the most common clinical finding across all age groups and virus variants, a result that is consistent with most of the availiable studies [10,22,23]. The most common hematological disorders were neutropenia (30%) and lymphopenia (23%), while the Omicron strain was associated with a longer length of hospitalization. Similar results have been found in other studies worldwide, even from the early stages of the pandemic, highlighting the consistency of our findings with those in the literature. While Omicron infections are generally considered milder than Alpha and Delta, our finding of longer hospital stays for Omicron may reflect several factors. One possible explanation is the significantly larger sample size in the Omicron group, which could include a wider range of cases, such as patients with milder symptoms or those with comorbidities requiring extended monitoring. Furthermore, it is worth mentioning that, since the specific viral variant was not determined for individual patients, it cannot be confirmed that all children were infected with the predominant variant of the respective sub-period, given that variant prevalence was not absolute. There are several overlapping periods in variant circulation. For instance, in January 2022, Delta and Omicron were both highly prevalent. As a result, some cases classified as Omicron infections might have actually been caused by the Delta variant. Given Delta’s known aggressiveness, the observed longer hospitalization times in children attributed to Omicron infections could potentially be explained by unrecognized Delta cases.

A 2020 systematic review and meta-analysis in China reported that 84% of children with COVID-19 had mild or moderate disease, with fever and cough being the predominant symptoms [10]. On the contrary, in a very recent—2024—global systematic review of the epidemiology and clinical features of SARS-CoV-2 infection in in the pre-Omicron era, it was estimated that about 20.7% of people aged ≤18 years who were positive for SARS-CoV-2 required admission to the hospital, but only 6.05% had severe or critical disease [23]. A similar 2022 Greek study involving 2971 pediatric patients showed that fever was the most common symptom (60.3%), followed by cough (47.4%), runny nose (21.1%), and diarrhea (10.3%). Most children (>90%) recovered completely without complications. Fever appears to be the most common symptom of SARS-CoV-2 infection, with the highest frequency observed in infection with the Omicron strain, as was the case in our study [24]. Regarding anosmia and/or ageusia—symptoms observed frequently in adults—studies suggest that they are less common in children but may serve as strong predictors of SARS-CoV-2 infection [15].

A previous review from our center, which analyzed data from multiple studies, found that most children with COVID-19 had normal leukocyte counts. Among white blood cell abnormalities, leukopenia was the most commonly observed. Age appeared to play a role, as neonates and infants more commonly displayed lymphocytosis as the predominant abnormality [25]. Lymphopenia, which is often found in adult patients, is not as common in children, with most studies reporting an incidence of around 15%. This difference may be attributed to the immaturity of the pediatric immune system or the generally milder course of COVID-19 in younger populations [25]. In our study, the majority of children had a normal leukocyte count, and the most common hematological disorders were neutropenia (30%) and lymphopenia (23%). In infants and children up to 8 years of age, the most common manifestation was neutropenia (28%), while in adolescents, lymphopenia was more frequent (43%)—results that align with other studies in pediatric populations [26]. In correspodence, a 2023 study from South Korea showed that more children <2 years of age and adolescents aged 10 to <19 years experienced neutropenia and lymphopenia, respectively, during the Delta wave [26]. Neutropenia, as a consequence of viral infections, is caused by the formation of antineutrophil antibodies, cytokine production, or bone marrow suppression, and its severity is usually mild to moderate [27]. A retrospective study similar to ours found that an increase in monocytes was the most frequent hematological finding, while a reduced number of eosinophils and an increased neutrophil-to-lymphocyte ratio could be used as prognostic indicators of severe infection [28].

Irregularities involving red blood cells and platelets have been shown to be relatively rare among the children [25]. Regarding the blood coagulation system, it is well known that COVID-19 in adults is a prothrombotic state that disrupts the coagulation pathway [29,30,31]. In the pediatric population, observational studies have shown that it can also induce deep vein thrombosis, but the data remain incomplete [31,32]. The International Society on Thrombosis and Hemostasis recommends prophylactic administration of low-molecular-weight heparin (LMWH) in children with COVID-19 who have significantly elevated D-dimer levels or clinical risk factors for thrombophilia [33]. In the present study, no significant coagulation disorders were observed, except for a mild transient prolongation of aPTT in 2% of children and elevated D-dimer levels (>1) in 15%. A multicenter prospective cohort study in Canada showed that D-dimers and fibrinogen are most frequently affected, while platelets usually remain at normal levels in children [31]. However, in contrast to our population, most studies conducted coagulation mechanism tests only in severe cases or in patients with risk factors [30,32,34]. Thrombotic episodes in children appear to be uncommon, mainly involving serious infections or MIS-C [35,36]. A large systematic review of 48.322 children with COVID-19 showed that 34 (27%) studies reported thrombotic complications in 504 patients (1.04%), while 31 (25%) studies reported bleeding complications in 410 patients (0.84%) [37]. A large multicenter study in the USA found that the risk of children with MIS-C developing deep vein thrombosis is approximately 4%, much lower than the 21% observed in adults [36].

Chest X-rays in children diagnosed with COVID-19 are often unremarkable or may reveal non-specific changes typically associated with viral pneumonia [38], such as in our population. However, more detailed imaging studies, such as chest CT scans, have provided additional insights into pulmonary involvement in pediatric cases. For example, a study conducted by Lu et al. found that 56% of pediatric patients showed ground-glass opacities, which are a hallmark feature of viral lung infections, on their chest CT scans [39]. In another investigation, Cui et al. evaluated a larger cohort of children and provided further evidence supporting these findings, expanding the understanding of imaging characteristics in this population [40].

In general, children tend to experience milder symptoms compared with adults, with fatalities being exceedingly rare [41]. Young age and the presence of underlying health conditions are often cited as potential risk factors for severe COVID-19 in children, though not all studies have consistently confirmed these associations [41]. When considering comorbidities and risk factors for severe disease, a large multicenter prospective study in Germany showed that the most common comorbidities in hospitalized children with COVID-19 were obesity, diabetes, and neurological/neuromuscular disorders, followed by cardiovascular and respiratory diseases. Most deaths during active infection (15/18) involved comorbidities. This aligns with our findings, in which the fatal case involved a child with Sanfilippo syndrome [42,43]. No correlation was found between deaths and the prevailing mutation. Similarly, a large cross-sectional study in the USA involving 43,465 patients showed that the strongest risk factors for hospitalization are diabetes mellitus and obesity, while those for the development of severe disease are diabetes mellitus and congenital cardiac/vascular anomalies [44,45].

Regarding SARS-CoV-2 variants, most studies have not identified a strong association between a mutation and specific clinical findings [43]. A recent study indicated that anosmia was more common in Delta-variant infections, while vomiting, diarrhea, fever, neck pain, and lymphopenia predominated in Omicron variant cases [46]. A large multicenter cohort study in Germany showed that, during the Omicron wave, hospitalizations increased only in the infant age group, but the percentage of those requiring treatment did not, suggesting that this may have been due to the greater spread of the virus but also to preventive measures by the treating physicians [35]. Regarding the risk of ICU admission, a study from Qatar showed that the 12–17-year-old age group had a greater overall impact, while, for the Omicron mutation, it was the the 1–4-year-old age group, perhaps indicating a downward shift in the age spectrum during the Omicron wave. The specific study also came to the conclusion that Omicron-variant infection in children/adolescents is associated with less severe disease than Delta-variant infection as measured by hospitalization rates and the need for ICU care or mechanical ventilation [47].

MIS-C was a relatively rare occurrence within our study population, a finding that aligns with the previously published literature [19]. Studies have consistently documented that the most common presenting symptoms of MIS-C include persistent fever and gastrointestinal disturbances such as abdominal pain, vomiting, and diarrhea, along with dermatological manifestations like rash and ocular symptoms such as conjunctivitis. Typically, children present with fever lasting three to five days, which is often followed by the development of shock and/or the involvement of multiple organ systems [48]. In our study, specific hematological abnormalities were observed among children diagnosed with MIS-C. Lymphopenia was noted in 50% of cases, anemia in 75%, and thrombocytopenia in 25%. These findings are consistent with laboratory abnormalities reported in the literature, which frequently include lymphopenia, elevated inflammatory markers such as CRP and D-dimer, and increased cardiac markers indicative of cardiac stress or inflammation [49,50]. The management of MIS-C generally involves a combination of intravenous immune globulin (IVIG) and glucocorticoids, which are the cornerstone of treatment as per the current clinical guidelines. Children with MIS-C are also recognized to have an elevated risk of thrombotic events, and, for this reason, low-dose aspirin is commonly prescribed as a preventive measure. At our center, all children diagnosed with MIS-C received treatment in line with these established guidelines, and all experienced positive outcomes without long-term complications. The prognosis for children with MIS-C is generally favorable, as supported by findings in the broader medical literature. Most children achieve a full clinical recovery with the appropriate medical intervention. A systematic review encompassing 16 case series and a total of 655 children with MIS-C found that the condition is associated with a low mortality rate; only 11 deaths were reported, accounting for 1.7% of the cases [51]. These findings underscore the importance of timely recognition and management of MIS-C to ensure optimal outcomes for affected children.

This study has several limitations, primarily due to its retrospective nature. Additionally, as it concerns data from a single center, the findings may not be generalizable. Finally, as in similar studies [42], the use of the predominant variant per time period may not accurately reflect all infections during a specific period, given the possible simultaneous circulation of different strains.

## 5. Conclusions

In conclusion, this study examined the hospitalization patterns and clinical characteristics of 254 pediatric COVID-19 cases, ranging in age from 5 days to 16 years. Fever was the most common symptom, followed by poor feeding and gastrointestinal symptoms. Hematological abnormalities, including neutropenia and lymphopenia, were observed, while coagulation issues were rare. By age group, fever was the most common symptom, with gastrointestinal issues more prevalent in younger children. Neutropenia was common in infants, while lymphopenia was more frequent in adolescents. The Omicron variant was the most prevalent and was associated with longer hospital stays and more gastrointestinal symptoms. No significant differences in laboratory findings were noted across the different SARS-CoV-2 variants. These findings highlight the need for the careful monitoring and management of pediatric COVID-19 cases.

## Figures and Tables

**Table 1:** Demographic characteristics and underlying diseases by age group.

Age	N = 254	Male Sex	Underlying Disease
Infants	117	51.3%	2.6%
1–5 y.o.	59	55%	15%
6–11 y.o.	31	61%	16%
>12 y.o.	47	57%	1%

**Table 2:** Clinical manifestations by age group.

Age Group	Fever	Cough	Respiratory Distress	Reduced Feeding	Gastrointestinal Tract Symptoms
Infants	80.6%	27.7%	9.5%	36.1%	32.5%
1–5 y.o.	88%	55%	13%	47%	43%
6–11 y.o.	63%	41%	41%	42%	53%
>12 y.o.	78%	44%	2%	2%	57%

**Table 3:** Laboratory findings by age group.

**Age** **Group**	**Leukopenia** **(%)**	**Neutropenia** **(%)**	**Lymphopenia** **(%)**	**Elevated Inflammatory Markers (%)**	**Abnormal Coagulation Studies (%)**
Infants	51%	25.6%	6.8%	7.7%	6%
1–5 y.o.	20%	28%	20%	33%	8%
6–11 y.o.	54%	35%	41%	25%	6%
>12 y.o.	57%	32%	51%	17%	10%
*p*-Values *	0.001	0.13	0.13	0.37	0.4

* The *p*-values represent the results of chi-square tests conducted across different age groups. Post hoc analyses, specifically pairwise chi-square tests between groups, were performed only for leukopenia, where the *p*-value was statistically significant, to identify the specific groups that differed. Leukopenia was statistically significantly less common in the age group 1 to 5 years old than in the other 3 groups (*p* < 0.05).

**Table 4:** Laboratory findings across SARS-CoV-2 strains.

Time Period	Virus Strain	N = 254	Leukopenia	Neutropenia	Lymphopenia	Duration of Hospitalization (Days)
1/5/2020–31/12/2020	Wuhan (wild type)	2%	16%	33%	33%	9
1/1/2021–30/7/2021	Alpha	9%	8%	20%	8%	5
1/8/2021–31/12/2021	Delta	17%	37%	23%	18%	4
1/1/2022–1/3/2023	Omicron	71%	1%	31%	24%	10
*p*-Values			0.7 *	0.7 *	0.7 *	0.024 **

* The *p*-values represent the results of chi-square tests conducted across different age groups. Post hoc analyses were not conducted since there was not a statistically significant result. ** The *p*-value represents the result of the Kruskal–Wallis test assessing the median duration of hospitalization across different time periods. Post hoc analysis using the Mann–Whitney test was conducted between the groups, revealing a statistically significant longer duration of hospitalization for the Omicron and Wuhan strains than that for the Alpha and Delta strains.

## Data Availability

Data are contained within the article.

## Abbreviations

WHO	World Health Organization
MIS-C	Multisystem inflammatory syndrome in children
